# The Role of the CXCL12/CXCR4/CXCR7 Chemokine Axis in Cancer

**DOI:** 10.3389/fphar.2020.574667

**Published:** 2020-12-08

**Authors:** Yi Shi, David J. Riese, Jianzhong Shen

**Affiliations:** Department of Drug Discovery and Development, Harrison School of Pharmacy, Auburn University, Auburn, AL, United States

**Keywords:** C-X-C motif chemokine ligand 12, C-X-C chemokine receptor type 4, C-X-C chemokine receptor type 7, cancer progression, tumor microenvironment

## Abstract

Chemokines are a family of small, secreted cytokines which regulate a variety of cell functions. The C-X-C motif chemokine ligand 12 (CXCL12) binds to C-X-C chemokine receptor type 4 (CXCR4) and C-X-C chemokine receptor type 7 (CXCR7). The interaction of CXCL12 and its receptors subsequently induces downstream signaling pathways with broad effects on chemotaxis, cell proliferation, migration, and gene expression. Accumulating evidence suggests that the CXCL12/CXCR4/CXCR7 axis plays a pivotal role in tumor development, survival, angiogenesis, metastasis, and tumor microenvironment. In addition, this chemokine axis promotes chemoresistance in cancer therapy via complex crosstalk with other pathways. Multiple small molecules targeting CXCR4/CXCR7 have been developed and used for preclinical and clinical cancer treatment. In this review, we describe the roles of the CXCL12/CXCR4/CXCR7 axis in cancer progression and summarize strategies to develop novel targeted cancer therapies.

## Introduction

Chemokines are small secreted peptides with molecular weights in the range of 8–12 kD (Rollins, 1997). They are best known for their roles in the mediation of immune cell recruitment (Rot and Von Andrian, 2004). Subsequently, they were reported to play essential roles in various pathological conditions, including inflammation, atherosclerosis, hematopoiesis, and cancer (Romagnani et al., 2004; Ma et al., 2013; Griffith et al., 2014; Wang et al., 2018). Based on the arrangement of the two cysteine residues near the amino terminus, chemokines can be classified into four subfamilies (CC, CXC, CX3C, and C) (Nomiyama et al., 2013). Chemokines exert their function by binding to seven-transmembrane- spanning G protein-coupled cell-surface receptors. Named by their endogenous ligand (chemokines), chemokine receptors are grouped into two subfamilies: conventional chemokine receptors (CCKRs) and atypical chemokine receptors (ACKRs) (Bachelerie et al., 2014). Chemokines binding to CCKRs would induce a conformational change in the receptor, leading to intracellular signal transduction (Kufareva et al., 2015). However, ACKRs do not couple to many signal transduction pathways, which are considered as scavengers for chemokines (Bachelerie et al., 2014).

Stromal cell-derived factor-1 (SDF-1), which is also referred to as CXCL12, is a homeostatic CXC chemokine that possesses seven different isoforms. It is secreted in a wide range of different tissues by stromal cells, fibroblasts, and epithelial cells, regulating hematopoietic cell trafficking and secondary lymphoid tissue architecture (Luther et al., 2002). Growing evidence has shown regulatory roles of the tumor stromal cell interactions in tumor initiation and progression (Barbieri et al., 2010). CXCL12 and its receptors, CXCR4 and CXCR7, have been identified as the key factors in tumor development and metastasis Ovarian epithelial tumor cells express high levels of CXCL12. CXCL12 induces signaling via the AKT and ERK pathways, stimulating ovarian cancer cell growth *in vitro* (Scotton et al., 2002). Similarly, the expression of CXCL12 is associated with pathological features and clinical outcomes in human breast cancer (Kang et al., 2005). Moreover, CXCL12 is expressed at high levels in bladder cancer (Yang et al., 2015a), gastric cancer (Ishigami et al., 2007), hepatocellular carcinoma (Ghanem et al., 2014), prostate cancer (Zhang et al., 2008), lung cancer (Imai et al., 2010), and many other human tumors (Sakai et al., 2012; Qiao et al., 2016; Teng et al., 2016). However, the exact functions of CXCL12 in most cancers are yet to be fully elucidated.

CXCR4, also known as fusin, is a highly conserved seven transmembrane-spanning G-protein coupled receptor. It consists of 352 amino acid residues, including an amino (N)-terminus, three extracellular and intracellular loops, 7 TM helices, and a carboxyl (C)-terminus (Wu et al., 2010). CXCL12 is the only confirmed chemokine that binds to CXCR4, although studies implicate other factors activate this receptor, such as macrophage migration inhibitory factor (MIF) and extracellular ubiquitins (Bernhagen et al., 2007; Saini et al., 2011). CXCR4 was initially found to function as a co-receptor required for entry of T-tropic (X4) HIV viruses that target CD4-positive T cells (Feng et al., 1996). Later, CXCR4 has been discovered strongly expressed in multiple types of cancers, including breast cancer (Müller et al., 2001), kidney cancer (Pan et al., 2006), ovarian cancer (Jiang et al., 2006), thyroid cancer (De Falco et al., 2007), prostate cancer (Hirata et al., 2007), lung cancer (Gangadhar et al., 2010), colon cancer (Lv et al., 2014), and thymoma (Wang et al., 2016). Along with its ligand CXCL12, CXCR4 controls the transduction of different downstream signaling pathways that are profoundly involved in tumor cell survival, proliferation, and migration (Kijima et al., 2002; Marchesi et al., 2004; Yang et al., 2019). In addition, increasing evidence showed that CXCR4 not only plays a marked role in cancer metastasis but also in cancer stem cells (Bagri et al., 2002; Kucia et al., 2005).

CXCR7, also named atypical chemokine receptor 3 (ACKR3), is a seven-transmembrane G-protein coupled receptor. It was originally cloned from a dog thyroid cDNA library and named receptor dog cDNA 1 (RDC-1) (Libert et al., 1989). CXCR7 was initially presumed to be a receptor for vasoactive intestinal peptide (VIP) (Sreedharan et al., 1991) and calcitonin gene-related peptide 1 (CGRP1) (McLatchie et al., 1998), but subsequent studies did not confirm this. CXCR4 has been thought to be the only receptor for CXCL12. However, CXCR7 was identified to be a higher-affinity receptor for CXCL12 than is CXCR4 in 2005 (Balabanian et al., 2005). CXCR7 has been regarded as a scavenger receptor for CXCL12 in some studies (Naumann et al., 2010), while other evidence suggested that CXCR7 could induce intracellular signaling associated with CXCR4 (Levoye et al., 2009). Current studies have found CXCR7 is present in diverse tumor cell lines, including breast cancer (Burns et al., 2006), cervical carcinoma (Burns et al., 2006), glioma (Hattermann et al., 2010), and pancreatic cancer (Liu et al., 2014). Additionally, the expression of CXCR7 is extensively high in the endothelial cells of tumor tissues (Miao et al., 2007). Another research group addressed a correlation between the expression of CXCR7 and enhanced adhesive/invasive activities in prostate cancer (Wang et al., 2008a). These specific features suggest that CXCR7 is closely related to tumor progression.

This review summarizes the downstream cell signaling transduction of CXCL12/CXCR4/CXCR7 axis (abbreviation: CXCL12 axis) and the role of CXCL12 axis in tumor progression, growth, survival, angiogenesis, metastasis, and chemoresistance. We also emphasize the therapeutic targeting of the CXCL12 axis for cancer treatment.

## C-X-C Motif Chemokine Ligand 12 Axis Signal Transduction

**FIGURE 1:**
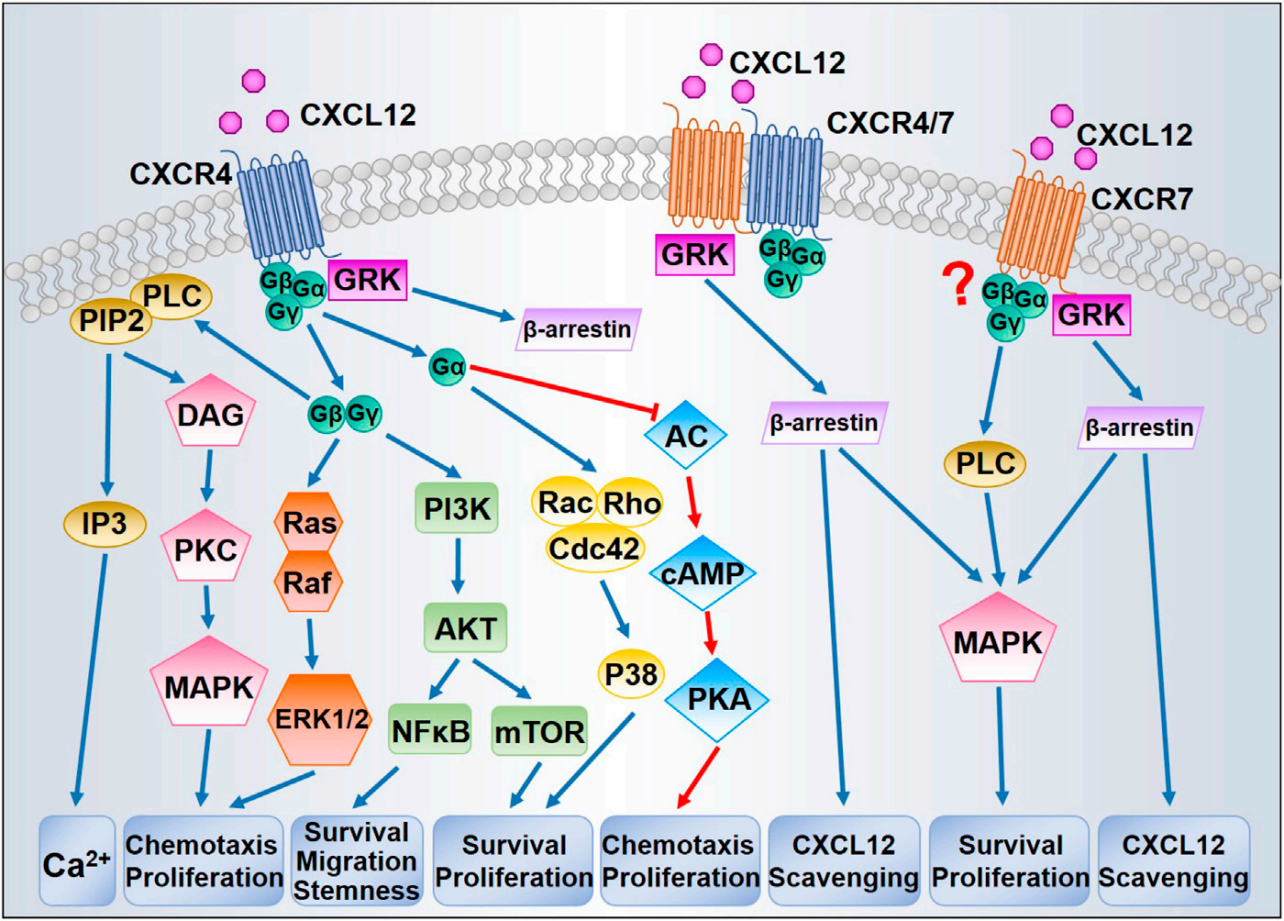
Proposed CXCL12/CXCR4/CXCR7 signaling pathways. After binding with CXCL12, CXCR4 activates downstream signaling through G proteins and GRKs. Dissociation of the G protein complexes subsequently triggers MAPK, ERK1/2, and AKT signaling pathways, thereby promoting cell survival and proliferation. GRKs mainly induce the recruitment of β-arrestin leading to CXCR4 internalization. CXCR7 could induce β-arrestin independently or through CXCR4/CXCR7 heterodimer, resulting in MAPK activation and CXCL12 scavenging. The question mark indicates that whether CXCR7 is coupled to G protein has been in debate.

CXCL12/CXCR4/CXCR7 can stimulate diversified downstream signaling pathways that regulate chemotaxis, gene transcription, cell survival, and proliferation. Figure 1 presents the principal signaling pathways thought to be involved in CXCL12 signal transduction. The precise transduction may differ between cell types as some features might be tissue-dependent.

CXCL12 binding to CXCR4 promotes a three-dimensional conformation change and initiates the exchange from GTP to GDP, leading to the dissociation of Gα subunit from Gβ/Gγ dimer (Bajetto et al., 2001). The dissociated Gβ/Gγ dimer is capable of activating phospholipase C (PLC)-β, which catalyzes the hydrolysis of phosphatidylinositol (4,5)-bisphosphate (PIP2) into two secondary messengers, inositol (1,4,5)- trisphosphate (IP3) and diacylglycerol (DAG). Upon binding with IP3, IP3 receptor (IP3R) triggers the release of calcium from intracellular stores into the cytoplasm (Mellado et al., 2001). DAG promotes the activation of protein kinase C (PKC) and mitogen-activated protein kinase (MAPK), which contributes to chemotaxis (Sun et al., 2002). The Gβ/Gγ dimer is additionally involved in Ras activation of the MAPK/ERK cascade, inducing profound consequences for gene expression and cell cycle progression (Würth et al., 2014). Based on the coupled Gα subunits, diverse GPCR signaling pathways can be classified into four families: Gα_s_, Gα_i_, Gα_q_, and Gα_12_ (Goldsmith and Dhanasekaran, 2007). At first, CXCR4 was classified as a Gα_i_-protein-coupled receptor (Gupta et al., 1998). The activation of Gα_i_ subunits inhibits adenyl cyclase, which catalyzes 5′adenosine triphosphate into cyclic adenosine monophosphate (cAMP), thereby regulating other downstream effectors (Gerits et al., 2008). Either the Gβ/Gγ dimer or the Gα_i_ subunit can activate phosphoinositide-3 kinase (PI3K), leading to phosphorylation of multiple focal adhesion proteins and contributing to cell migration (Wang et al., 2000). By generating phosphatidylinositol (3,4,5)- triphosphate, PI3K can trigger the activation of the serine-threonine kinase AKT, thus stimulating the downstream transcription factor nuclear factor- κB (NF-κB) and mTOR pathways, which play key roles in tumor cell survival and proliferation (Barbero et al., 2003; Ward, 2006). In addition, Gα_i_ is in some contexts necessary for Rac activation (Li et al., 2013). Despite the well-characterized coupling to Gα_i_, CXCR4 can also transduce signal through other Gα proteins, such as Gα_q_ and Gα_12/13_. CXCR4 coupled to Gα_q_ can couple to downstream events via PLC-β, which leads to increased IP3 synthesis and PKC signaling (Princen et al., 2003). CXCR4 stimulation of Gα_12/13_ can also stimulate Bruton’s tyrosine kinase, which inhibits Fas-mediated apoptosis (Jiang et al., 1998). Furthermore, Gα_12/13_ is required as a direct activator of Rho through the modulation of Rho-guanine nucleotide exchange factors (Rubin, 2009). CXCR4 signaling is modulated by receptor internalization and lysosomal degradation. Following CXCL12 binding, the intracellular C-terminus of CXCR4 is rapidly phosphorylated at serine sites by G-protein coupled receptor kinases (GRKs), resulting in recruitment of β-arrestin and clathrin-mediated endocytosis (Busillo and Benovic, 2007). β-arrestin prevents the CXCR4 from coupling with G proteins and targets them for lysosomal degradation (Luttrell and Gesty-Palmer, 2010).

Initially, CXCR7 was characterized as a scavenger or decoy receptor for CXCL12 due to the absence of typical intracellular responses (such as intracellular calcium mobilization or modulation of adenylyl cyclase activity) after CXCL12 binding (Zabel et al., 2009). Moreover, unlike CXCR4 internalization, CXCR7 internalization occurs even in the absence of ligand binding and does not result in receptor degradation (Klein et al., 2014). Like CXCR4, CXCR7 can activate numerous intracellular signaling pathways, especially the AKT and MAPK pathways, via G-proteins (Odemis et al., 2012) or by β-arrestin (Gravel et al., 2010). CXCR4 and CXCR7 can each form homo- and heterodimers (Luker et al., 2009). The formation of heterodimers enhances CXCL12-dependent intracellular calcium mobilization and ERK1/2 phosphorylation (Sierro et al., 2007), yet blocks CXCR4 coupling to G protein complexes (Levoye et al., 2009). Therefore, CXCR7 signal transduction is still under intense study, particularly with respect to mechanisms of signaling specificity.

## C-X-C Motif Chemokine Ligand 12 Axis in Tumor Progression

Chemokines and their receptors have long been associated with cancer progression (Vicari and Caux, 2002; Tanaka et al., 2005; Janssens et al., 2018). More recently, the chemokine CXCL12 and its cognate receptors (CXCR4 and CXCR7) have been shown to play central roles in cancer proliferation, angiogenesis, invasion, tumor microenvironment, as well as drug resistance induced by chemotherapy. There appear to be two mechanisms by which CXCL12 affects tumor cell biology: 1) direct stimulation of signaling pathways that promote cancer cell growth, metastasis, and angiogenesis; 2) indirect effects, including the recruitment of CXCR4/CXCR7-positive cancer cells to CXCL12-expressing organs (Duda et al., 2011).

### C-X-C Motif Chemokine Ligand 12 Axis in Tumor Cell Growth and Survival

**FIGURE 2:**
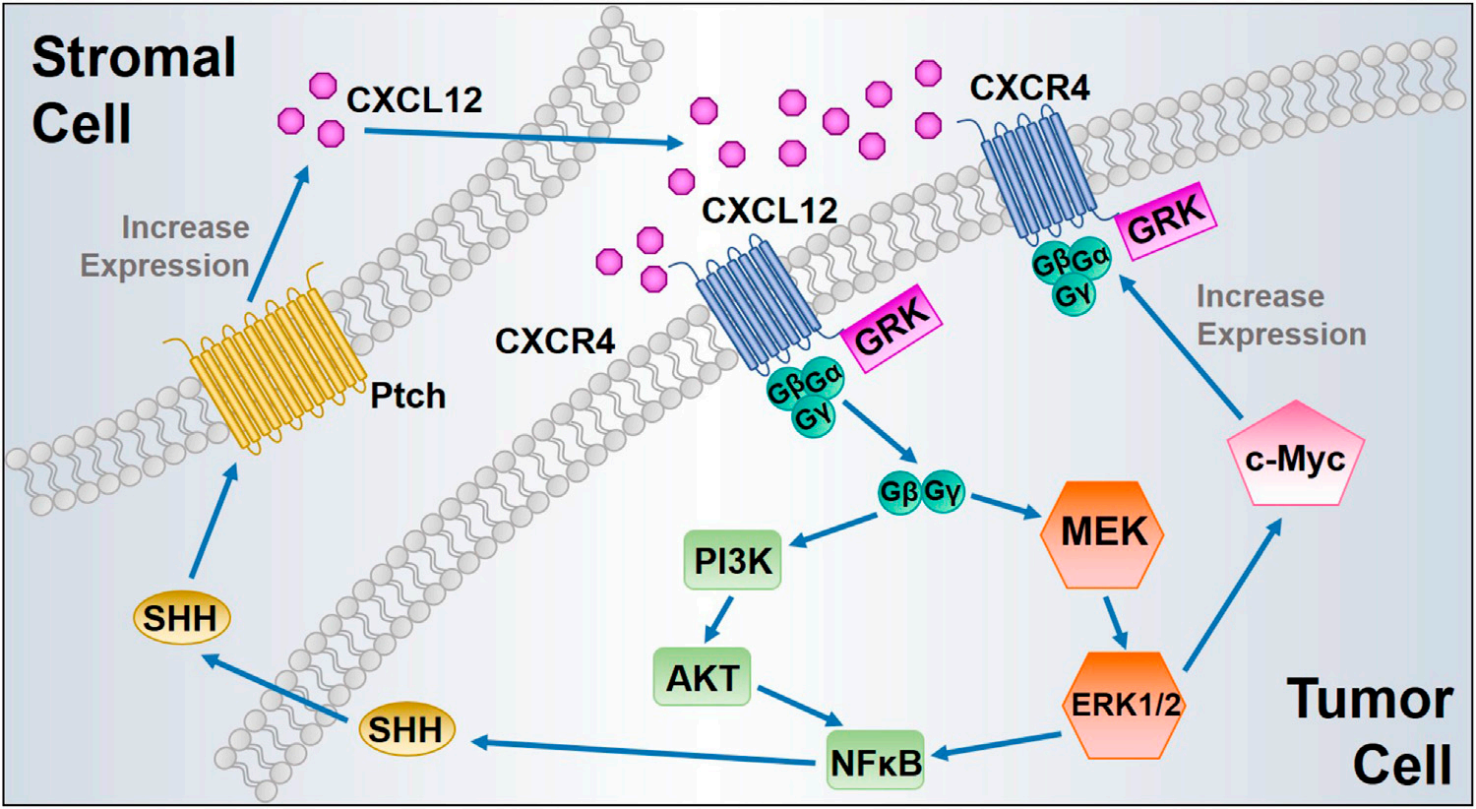
Proposed positive feedback loops of CXCL12/CXCR4 signaling to enhance tumor cell proliferation. ERK1/2 MAPK activated by CXCL12/CXCR4 interaction induces c-Myc signaling, leading to CXCR4 upregulation with increased cancer cell proliferation. In addition, CXCL12 binding to CXCR4 triggers NFκB signaling, which induces SHH synthesis and release from the tumor cells. Secreted SHH promotes CXCL12 upregulation and release from the stromal cells after its binding to protein patched homolog (Ptch), which in turn activates CXCL12/CXCR4 axis in tumor cells.

One of the major biological effects modulated by the CXCL12 axis is to promote tumor cell survival and proliferation. In 1998, Sehgal et al. first found CXCR4 was overexpressed in glioblastoma cell lines, and the expression of antisense CXCR4 inhibited glioma cell proliferation (Sehgal et al., 1998). Furthermore, exogenous CXCL12 induces proliferation in a dose-dependent manner in human glioblastoma cell lines (Barbero et al., 2003). The CXCL12 axis has been identified to induce proliferation of cell lines derived from many types of cancers, including prostate cancer (Fernandis et al., 2002), breast cancer (Ueda et al., 2006), lung cancer (Miao et al., 2007), multiple myeloma (Beider et al., 2011), and pancreatic cancer (Gao et al., 2010). The CXCL12/CXCR4 interaction phosphorylates CXCR4, subsequently promotes calcium flux, and directly activates MAPK, PI3K, Wnt, and Sonic Hedgehog signaling pathways, thus inducing proliferation of various types of tumor cells (Getts et al., 2014). The activated MAPK alters translation of mRNA and phosphorylates several other cellular proteins (c-Myc and RSK kinases) that are critical to cell proliferation, cell cycle progression, cell division, and differentiation. CXCR4 expression can be upregulated by the transcription factor c-Myc, which in turn activates MAPK. Therefore, CXCR4 expression and MAPK signaling form a positive feedback loop to further sustain proliferative signaling (Figure 2) (Thomas et al., 2008).

Activation of CXCR4 also increases expression of EGF/EGFR signaling proteins, leading to increased cell proliferation (Weekes et al., 2012). Wnt signaling plays a pivotal role in CXCL12-induced tumor cell proliferation, as silencing CXCL12/CXCR4 signaling influences pancreatic cancer cell phenotypes and inhibits tumor cell proliferation *in vitro* via inactivation of the canonical Wnt pathway (Wang et al., 2008b). Additionally, the activation of AKT and ERK signaling pathways by the CXCL12 axis promotes nuclear accumulation of NFκB and increases NFκB signaling by inducing the phosphorylation and destabilization of IκB-α, followed by SHH up-regulation (Singh et al., 2012). Moreover, SHH signaling exerts its paracrine effect mainly by activating protein patched homolog (Ptch) on the surrounding stromal cells and subsequently induces additional CXCL12 expression and extracellular release to complete a positive pro-proliferative feedback loop (Figure 2) (Sleightholm et al., 2017). CXCR7 activates the AKT signaling pathway and stimulates EGFR signaling, thereby increasing tumor cell proliferation and survival (Wang et al., 2008a; Singh and Lokeshwar, 2011). Moreover, CXCR7 overexpression and gene silencing in tumor cells collectively support the role of CXCR7 contribution to tumor growth (Miao et al., 2007; Meijer et al., 2008). However, study in neuroblastoma (NB) indicated that CXCR7 activation strongly reduced the NB cell growth through ERK1/2 cascade both *in vitro* and *in vivo* (Liberman et al., 2012). Likewise, CXCR7 has been found associated with suppressing tumor growth and migration in colon cancer (Heckmann et al., 2014). These findings reflect a controversial role of CXCR7 in tumor cell proliferation, which also indicates that the function of CXCR7 might be cell type-specific. Extensive studies of its role in different malignancies would be beneficial for achieving precision medicine.

The CXCL12 axis also indirectly exerts anti-apoptotic effects in tumor cells. As mentioned previously, the CXCL12/CXCR4 axis activates AKT and ERK, subsequently leading to NFκB accumulation, which can suppress apoptotic signaling (Ganju et al., 1998). The induction of MAPK-ERK and PI3K pathways by CXCL12 inactivates the pro-apoptotic BAD (Bcl2-associated agonist of cell death) protein (Suzuki et al., 2001). This may arise through ERK phosphorylation of Bad on serine 112, which results in the dissociation of Bad from Bcl-2 and enables Bcl-2 to exert its anti-apoptotic effects (Scheid et al., 1999). Similarly, the CXCL12/CXCR4 axis may stimulate ERK phosphorylation of Bim, resulting in Bim dissociation from the anti-apoptotic proteins Bcl-2 and Mcl-1 and enabling the free Bcl-2 and Mcl-1 to exert their anti-apoptotic effects by binding to Bax (McCubrey et al., 2007). CXCR7 signaling also suppresses apoptosis. CXCR7 overexpression reduces the apoptotic fraction in prostate cancer cells and protects these cells from apoptosis (Wang et al., 2008a). Likewise, knockdown of CXCR7 in the MCF7 breast cancer cell line increased the expression of the pro-apoptotic caspase three and eight proteins (Gao et al., 2015).

Another function of the CXCL12 axis in tumor cell growth and survival is evading growth suppression, which is most commonly regulated through the Rb or p53 pathways. Wildtype p53 binds to the GFI-1 binding site in the proximal enhancer region of the CXCR4 gene, which suppresses CXCR4 expression (Katoh and Katoh, 2010). Treatment with p53 rescue drugs (PRIMA-1, CP- 31398) in p53 mutant cells can restore the suppression of CXCR4 transcription in cells with mutant p53. Loss of functional p53 is commonly observed in many cancer cell lines, which is one of the mechanisms resulting in CXCR4 upregulation. As mentioned before, the CXCL12/CXCR4 axis increases the expression of pro-survival proteins MDM-2 and NFκB through AKT activation. Specifically, MDM-2 phosphorylation of p53 directly leads to its degradation through ubiquitin-dependent proteolysis, thereby promoting tumor cell survival and proliferation (Sleightholm et al., 2017).

### C-X-C Motif Chemokine Ligand 12 Axis in Angiogenesis

Both *in vitro* and *in vivo* studies suggest that the expression level of CXCL12/CXCR4 in cancer cells is positively correlated with microvessel density. Initially, the angiogenic activity of CXCR4 was inferred in mice lacking CXCL12 or CXCR4 (Tachibana et al., 1998). For example, mice lacking CXCR4 or CXCL12 are defective in the formation of the large vessels supplying the gastrointestinal tract and exhibit defects in vascular development, hematopoiesis, and cardiogenesis. Moreover, CXCR4 is highly expressed in the endothelial cells of large vessels in tumor stroma, indicating that the CXCL12/CXCR4 signaling plays a vital role in tumor angiogenesis (Hayashi and Kume, 2008). There are four possible mechanisms by which CXCL12/CXCR4 regulates tumor angiogenesis: 1) upregulates vascular endothelial growth factor (VEGF) expression in tumor tissue through the PI3K/Akt signaling pathway; 2) reduces the expression of glycolytic enzyme phosphoglycerate kinase 1 (PGK1) which suppresses the secretion of VEGF; 3) upregulates several angiogenesis-associated genes in cancer cells; and 4) directs the recruitment of endothelial progenitor cells to the vicinity of neovascularization.

Among various factors involved in tumor angiogenesis, VEGF and its receptor VEGFR play a major role (Carmeliet, 2005). VEGF can regulate angiogenesis indirectly by inducing endothelial cells to express MMP-2 and MMP-9, thereby enabling chemotaxis of endothelial cells and the formation of capillary channels, and thus indirectly regulate angiogenesis (Lafleur et al., 2003). Moreover, CXCR4/CXCL12 induces AKT phosphorylation, which can upregulate VEGF transcription and protein expression (Liang et al., 2007). Under hypoxic conditions, hypoxia-inducible factor 1 (HIF-1) and VEGF increase the expression of CXCR4 in human brain microvascular endothelial cells, which promotes glioblastoma angiogenesis (Zagzag et al., 2006). CXCL12 can induce MMP-2 and MMP-9 upregulation in pancreatic cancer cells (Pan et al., 2013). Phosphoglycerate kinase 1 (PGK1) is an ATP-generating glycolytic enzyme that catalyzes the reversible transfer of a phosphate group from 1,3-bisphosphoglycerate (1,3-BPG) to ADP, producing 3-phosphoglycerate (3-PG) and ATP. PGK1 is secreted extracellularly by different types of tumors, acting as a disulfide reductase that serves to cleave plasminogen, thereby generating the tumor blood vessel inhibitor angiostatin (Chen et al., 2003; Daly et al., 2004; Hwang et al., 2006). High levels of CXCL12 signaling through CXCR4 reduces PGK1 expression and promote angiogenesis (Wang et al., 2007). Another mechanism by which CXCL12 contributes to tumor angiogenesis is through the upregulation of angiogenesis-associated genes, among which IL-6 is the earliest and highest upregulated gene. For example, CXCL12 induces time- and dose-dependent upregulation of IL-6 transcription and protein secretion. This transcriptional regulation of IL-6 by CXCL12 is mediated by phosphorylation of ERK and activation of the NFκB complex (Chu et al., 2009). IL-6 induces other angiogenic factors, such as VEGF, basic fibroblast growth factor (bFGF), and COX-2 (Jee et al., 2004). Endothelial progenitor cells (EPCs) are pluripotent stem cells with the potential to differentiate into mature endothelial cells. Thus, EPCs play a pivotal role in tumor angiogenesis. In a coimplantation xenograft model, carcinoma-associated fibroblasts (CAFs) extracted from human breast carcinomas promoted the growth of admixed breast carcinoma cells by recruiting EPSs into the tumors (Orimo et al., 2005). Furthermore, CAFs secrete CXCL12 and the recruitment of EPCs is regulated in part by CXCL12. Moreover, CXCR4 is expressed on EPCs, thereby mediating CXCL12 signaling (Qin et al., 2016). Finally, the CXCL12/CXCR4 axis increases progesterone-induced EPC viability through the PI3K/AKT pathway (Yu et al., 2016). Plasmacytoid dendritic cells (DCs), which induce neoangiogenesis through production of IL-8 and TNF-α, could be attracted to the tumor environment by CXCL12 (Curiel et al., 2004). Extensive CXCR7has been observed in diverse tumor-associated blood vessels (Sánchez-Martín et al., 2011) and could be upregulated in endothelial cells by hypoxia (Bosco et al., 2006). The immunohistochemical staining results demonstrated that CXCR7 was widely expressed in human breast and lung cancers, where it was highly expressed on a majority of tumor-associated blood vessels and malignant cells but not expressed on normal vasculature (Miao et al., 2007). Downregulation of CXCR7 expression by siRNA resulted in the formation of smaller tumors by these cells. These results are consistent in clinical biopsy samples of ovarian cancer, bladder cancer, kidney cancer, and malignant gliomas (Madden et al., 2004). CXCR7 overexpression in murine breast cancer cells promotes tumor development by enhancing angiogenesis (Hernandez et al., 2011). In addition, it was observed in human PCa cells that CXCR7 regulates the expression of the proangiogenic factors interleukin-8 or VEGF, which participates in the regulation of tumor angiogenesis (Wang et al., 2008a).

### C-X-C Motif Chemokine Ligand 12 Axis in Invasion and Metastasis

Metastasis is an important biological characteristic of malignant tumors, which is the key cause of death among cancer patients. Tumor metastasis was once recognized as a passive consequence of a single tumor cell escaping from a primary tumor. However, recent data indicated that tumor metastasis is an active process employing multiple molecular and cellular mechanisms (Chambers et al., 2002). The CXCL12 axis is also involved in metastasis of many human cancers, such as pancreatic cancer (Wang et al., 2008b), melanoma (Bartolomé et al., 2009), and colon cancer (Zeelenberg et al., 2003).

In 2001, Muller et al. provided the first evidence that the CXCL12/CXCR4 axis mediates human breast cancer metastasis (Müller et al., 2001). For example, a CXCR4 neutralizing antibody and shRNA knockdown of the CXCR4 receptor significantly reduced tumor cell invasion (Krohn et al., 2009). Moreover, CXCL12 is highly expressed in liver and specifically attracts melanoma and CXCR4 (+) cells, thereby increasing cancer liver metastasis (Kim et al., 2006). Upon further study, the CXCL12/CXCR4 axis was shown to regulate metastasis via different mechanisms. Epithelial-to-mesenchymal transition (EMT) has been recognized as an important process that is associated with cancer metastasis. CXCL12/CXCR4 signaling stimulated the SHH signaling pathway, which is associated with EMT and loss of cell adhesion (Li et al., 2012). Moreover, CXCL12/CXCR4 signaling upregulates survivin via the MED/ERK and PI3K/AKT pathways, giving rise to cell cycle progression and EMT in human sacral chondrosarcoma (Yang et al., 2015b). Analogous findings were observed in glioblastoma (Liao et al., 2016) and hepatocellular carcinoma (Li et al., 2014). Moreover, CXCL12/CXCR4 signaling stimulates invasion and EMT of colorectal cancer cells through the Wnt/β-catenin signaling pathway (Hu et al., 2014). Metalloprotease (MMPs) are a family of enzymes involved in the degradation of extracellular matrix in the surrounding normal tissue with the ability to mediate cancer invasion and metastasis (Egeblad and Werb, 2002). CXCL12 promotes invasion of bone by myeloma cells by stimulating MMP-9 and MT1-MMP expression (Parmo-Cabañas et al., 2006). Similarly, CXCR4 promotes the migration and invasion by tongue squamous cell carcinoma cells by stimulating MMP- nine and MMP-13 expression through signaling by the ERK pathway (Yu et al., 2011). CXCL12/CXCR4 axis can stimulate the MMP-2 secretion of other types of cells; for example, CXCL12/CXCR4 signaling stimulates the migration of neuroblasts along the corpus callosum (Mao et al., 2016). Tumors are often hypoxic, resulting in upregulation of CXCL12 expression in endothelial cells by HIF-1. Therefore, CXCR4-positive cancer stem cells are likely to be attracted to the peripheral vessels, thereby serving as a pool for metastasis (Ratajczak et al., 2006). CXCL12 also modulates the expression and function of cell surface integrins, thereby promoting tumor cell adhesion. For example, CXCL12 increases the adhesion of PC- three cells to the human umbilical vein endothelial cell monolayer in a model of tumor extravasation or intravasation (Kukreja et al., 2005). Moreover, CXCL12 stimulates the expression of α5 and β3 integrins by prostate tumor cells (Engl et al., 2006), thereby inducing the adhesion of the tumor cells to human endothelium or extracellular matrix. Similarly, CXCL12 induces integrin β1 expression by ovarian tumor cells, leading to increased adhesion of these cells to laminin (Shen et al., 2009).

It has been postulated that CXCR7 expression is associated with increased tumor cell adhesion, which provides these tumor cells with a growth and survival advantage (Burns et al., 2006). Indeed, overexpression of CXCR7 enhances breast cancer cell adhesion to human umbilical vein endothelial cells (HUVECs). Likewise, increased CXCR7 expression is associated with increased prostate cancer cell aggressiveness; this effect appears to be mediated by cell adhesion proteins CD44 and cadherin-11 (Wang et al., 2008a). Collectively, these data suggest that CXCR7 functions as an oncogene, although much remains to be elucidated, particularly with respect to mechanisms of CXCR7 oncogenic signaling. CXCR7 deficient mice exhibit greater local recurrence of breast cancer following resection, suggesting that CXCR7 may possess breast cancer tumor suppressor activities related to the metastatic cascade (Stacer et al., 2016). This apparent conundrum may reflect the fact that CXCR7 can heterodimerize with CXCR4 and that loss of CXCR7 may disrupt the balance between oncogenic CXCR4/CXCR7 heterodimers and tumor suppressor homodimers. Clearly, this apparent dichotomy regarding CXCR7 function is yet to be definitively resolved.

### C-X-C Motif Chemokine Ligand 12 Axis in Tumor Microenvironment

The importance of the microenvironment to tumor progression is well established (Horgan et al., 1987; Barcellos-Hoff and Ravani, 2000; Bhowmick et al., 2004). The tumor microenvironment (TME) consists of resident non-cancerous cells (stromal fibroblasts, endothelial cells, and immune cells), proteolytic enzymes, growth factors, inflammatory cytokines, and the extracellular matrix (ECM) (Spill et al., 2016). CXCL12 regulates tumor-TME interactions, thereby promoting tumor survival, proliferation, angiogenesis, and metastasis (Burger and Kipps, 2006).

CXCL12 secreted by carcinoma-associated fibroblasts (CAFs) stimulates tumor growth directly, acting through CXCR4 expressed by breast cancer cells and promoting invasiveness (Orimo et al., 2005). CXCL12 also functions as a chemoattractant during tumor development. For example, CXCR4-positive cancer cells can be recruited to organs with high expression of CXCL12, including liver, lungs, and bone marrow (Wang et al., 2008b; Konopleva and Jordan, 2011). CXCR4 activation induces leukemia cell trafficking and homing to the bone marrow microenvironment because of the constitutive secretion of CXCL12 by stromal cells in bone marrow (Burger and Peled, 2009). At the same time, CXCL12 can attract CXCR4-positive inflammatory, vascular, and stromal cells into the tumor mass to support tumor development. This is a major contribution, as CXCR4 is expressed by many cell types in TME, including endothelial cells, epithelial cells, and lymphocytes (Burger and Kipps, 2006). It has been suggested that CAFs promote angiogenesis by recruiting endothelial progenitor cells (EPCs) into carcinomas, which is mediated by CXCL12 (Orimo et al., 2005). *In vivo* studies demonstrated that activation of CXCL12/CXCR4 accelerates the recruitment of fibroblasts and facilitates cancer stromal formation (Katoh et al., 2010). In a recent study, CXCL12 produced by both the multiple myeloma (MM) cells and bone marrow stromal cells (BMSCs) was found to regulate monocyte migration (Beider et al., 2014). And the blockage with anti-CXCR4 antibodies caused significant inhibition of monocyte recruitment. Monocytes differentiate into macrophages that support tumor cell proliferation, angiogenesis, and shape the immunosuppressive microenvironment. Besides, CXCL12 can trigger anti-apoptotic and proliferative signals in colon cancer cells by inducing mononuclear phagocytes to release HB-EGF, which binds the Epidermal Growth Factor Receptor (EGFR/HER1) and stimulates its signaling (Rigo et al., 2010). In addition, the stroma cells from specialized microenvironments are capable of modulating CXCR4 expression. For example, CFA secretion of transforming growth factor-β (TGF-β) potentiates CXCR4 stimulation of AKT signaling in human prostate epithelial cells, which indicates that synergism between TGF-β, CXCL12, and CXCR4 in tumor stroma contributes to carcinogenesis (Ao et al., 2007).

A recent study reported that CXCR7 is highly expressed on a majority of tumor-associated blood vessels (Miao et al., 2007). Moreover, CXCR7 expression is upregulated in human microvascular endothelial cells under hypoxic and acidic pH conditions, which are well-known characteristics of the TME (Monnier et al., 2012). Another work indicates that CXCR7 is also involved in TGF-β induced EMT in lung adenocarcinoma (Wu et al., 2016). Furthermore, regulation of the macrophage colony-stimulating factor/macrophage colony-stimulating factor receptor signaling pathway enables CXCR7 to recruit tumor-promoting macrophages to the tumor site (Wani et al., 2014). Finally, bone marrow microenvironment is necessary for CXCR7 activation, thereby promoting osteosarcoma invasion (Han et al., 2017). Altogether, these results strongly indicate that CXCR7 modulates cancer survival and metastasis via novel pathways involved in the tumor microenvironment.

### Transcriptional Regulation of C-X-C Motif Chemokine Ligand 12 Axis

In addition to the biological effects mediated by CXCL12 axis, investigations are also conducted associated with the transcriptional regulation of CXCL12 axis during cancer progression. Chen et al. reported that c-Myb could elevate CXCL12 expression by activating CXCL12 promoter in breast cancer cells (Chen et al., 2010). In pancreatic stellate cells, Galectin-1 was observed to induce CXCL12 secretion by activating NF-κB signaling pathway, thereby increasing pancreatic cancer metastasis (Qian et al., 2017). Recent work indicated that c-Myc regulates pancreatic cancer progression via HIF-1α/CXCL12/CXCR4signaling pathway (Liu et al., 2020). Knocking down of c-Myc significantly decreased CXCL12 expression and inhibited the invasion of pancreatic cancer cells. Moreover, the activating transcription factor 3 (ATF3) and the c-Jun dimerization protein2 (JDP2) inhibit CXCL12 secretion in tumor-associated fibroblasts in a lung carcinoma murine model (Avraham et al., 2019). NF-κB is one of the major transcription factors that regulate CXCR4 expression (Zhi et al., 2015). Further study revealed that elevated levels of NF-κB O-GlcNAcylation promoted CXCR4 expression in cervical cancer cells, thereby increasing lung metastasis (Ali et al., 2017). Furthermore, other transcription factors have also been suggested to increase mRNA and protein of CXCR4 in cancer development, including Runt-related transcription factor 2 (RUNX2) (Guo et al., 2016) and POU1F1transcription factor (Pit-1) (Martinez-Ordoñez et al., 2018). Similarly, overexpression of RUNX2 can induce CXCR7 transcription in prostate cancer (Bai et al., 2019). Another study demonstrated that CXCR7 expression had been upregulated by interleukin 6 (IL6) that is mainly derived from cancer-associated fibroblasts, contributing to chemoresistance in esophageal squamous cell carcinoma (Qiao et al., 2018).

## C-X-C Motif Chemokine Ligand 12 Axis Promotes Chemoresistance

The CXCL12 axis can contribute to tumor chemoresistance. For example, cancer chemotherapy upregulates CXCL12 and CXCR4 expression in multiple cancers (Shaked et al., 2008; Kioi et al., 2010). Moreover, patients who developed distant recurrence of rectal cancers exhibited much higher expression of both CXCR4 and CXCL12 than did patients who did not develop distant recurrence (Saigusa et al., 2010). A more direct evaluation of the role that the CXCL12 axis plays in chemoresistance indicates that the upregulation of CXCR4 in non-small cell lung carcinoma (NSCLC) mediates Gefitinib-resistance associated with EMT (Hu et al., 2017). Furthermore, the cancer progenitor population can be maintained by CXCR4 in tamoxifen-resistant breast cancer MCF7 cells by inducing AhR signaling (Dubrovska et al., 2012). CXCL12 enhances the resistance of chronic myeloid leukemia to adriamycin (ADM) by stimulating the expression of CXCR4. The mechanism features activation of the downstream PI3K/AKT pathway, translocation of NFκB dimers into the nucleus, and subsequent decrease of the expression of apoptosis-related proteins (Wang et al., 2014). Similarly, resistance to ADM can be partially reversed by CXCR4 silencing, and lapatinib-resistant cells exhibit greater CXCR4 expression than parental (sensitive) cells (De Luca et al., 2014). This chemoresistance is thought to be mediated by Src and CXCR4 signaling, particularly because CXCR4 antibody treatment reduces the invasive ability of cancer cells. Synthetic Exosome-Like Nanoparticles (SELNs) has been demonstrated to evoke apoptosis of human pancreatic cancer. However, further investigation indicated that SELNs induce the activation of NFκB, the expression and secretion of CXCL12, and stimulation of CXCR4/AKT survival pathway, resulting in protection of these tumor cells from death (Beloribi-Djefaflia et al., 2015). As mentioned before, CAF-secreted IL-6 stimulates the upregulation of CXCR7 through STAT3/NFκB signaling, promoting resistance of esophageal squamous cell carcinoma cells against cisplatin and 5-FU (Qiao et al., 2018). Taken together, these findings illustrate the contribution of CXCL12 axis to cancer chemoresistance.

## Therapeutic Targeting C-X-C Motif Chemokine Ligand 12 Axis

The CXCL12/CXCR4/CXCR7 axis is a potential target for cancer therapies. Up to now, several molecules have been developed to target CXCL12, CXCR4, or CXCR7. A deep understanding of CXCL12 axis in therapeutic applications would be beneficial for future translation of CXCL12, CXCR4, and CXCR7 inhibitors into clinical use.

### Prognostic Marker

Crowther-Swanepoel et al. first reported that functional coding mutations in CXCR4 might contribute to familial chronic lymphocytic leukemia (Crowther-Swanepoel et al., 2009). Similarly, genotyping 466 acute myeloid leukemia patients and 460 healthy controls indicates that a polymorphism in rs2228014 in the CXCR4 coding sequence is significantly increased in AML patients relative to healthy controls (Zheng et al., 2016). Moreover, CXCL12 and CXCR4 polymorphisms appear to contribute to increased risk of hepatocellular carcinoma (HCC) and may be potential markers for HCC (Chang et al., 2009; Qin et al., 2018). CXCR4 expression in triple-negative breast cancer (TNBC) cells correlates positively with histopathological grade but negatively with lymph node metastasis (Guembarovski et al., 2018). Moreover, heterozygosity for either CXCL12 and CXCR4 variants increases the risk for TNBC by analyzing genetic polymorphisms in 59 TNBC patients and 150 healthy control women. Similarly, it appears that a CXCR4 rs2228014 polymorphism is significantly associated with poor progression-free survival (PFS) in metastatic colorectal cancer patients (Matsusaka et al., 2017). Although CXCR4 expression is a prognostic factor in several human tumor types, none of the CXCL12/CXCR4/CXCR7 has yet been definitively validated as a tumor driver. Nonetheless, studies to date indicate that the CXCL12 axis is a tumor promoter rather than a tumor initiator.

### Preclinical Studies of Inhibitors

CTCE-9908 is a small peptide CXCL12 analog with CXCR4 antagonist activity. Treatment of osteosarcoma cells *in vitro* with CTCE-9908 causes decreased adhesion, migration, invasion, and growth rate (Kim et al., 2008). These *in vitro* effects are also observed *in vivo*, as mice treated with CTCE-19908 exhibit a 50% reduction in lung metastasis caused by tail vein injection of osteosarcoma cells. Similar effects are observed in other types of cancer cells, as CTCE-9908 inhibits ovarian cancer cell migration and induces multinucleation, G2-M arrest, and abnormal mitosis (Kwong et al., 2009). The anti-tumor and anti-metastatic effects of CTCE-9908 are dramatically enhanced by docetaxel in a breast cancer mouse model system (Hassan et al., 2011). Another approach for targeting the CXCL12 axis is the CXCL12 PEGylated mirror-image l-oligonucleotide (olaptesed-pegol). *In vivo*, olaptesed-pegol neutralize CXCL12, leading to a bone marrow microenvironment that is less receptive for multiple myeloma cells and reduces multiple myeloma cell homing and growth (Roccaro et al., 2014). AMD3465 is also a selective small-molecule CXCR4 antagonist that antagonizes CXCL12 stimulation of chemotaxis and prosurvival signaling pathways in leukemia cells (Zeng et al., 2009). *In vivo*, subcutaneous injections of BKT140 (a new-generation peptide CXCR4 inhibitor) significantly reduced the growth of human acute myeloid leukemia in a dose-dependent manner (Beider et al., 2011). Another CXCR4 antagonist, POL5551, exhibits inhibitory effect on glioblastoma growth and dissemination induced by anti-VEGF therapy (Gagner et al., 2017).

CCX733 is a selective CXCR7 antagonist that reduces the antiapoptotic effects of CXCL12 in glioma cells (Hattermann et al., 2010) and CXCR7-induced EMT in bladder cancer (Hao et al., 2012). The CXCR7 antagonists anti-CXCR7-12G8 and CCX771 both inhibit mTOR activation in renal cell carcinoma cells, resulting in reduced metastasis (Ierano et al., 2014). Similarly, the CXCR7 antagonists CCX754 and CCX771 inhibit lung metastasis of colorectal cancer cells *in vivo* (Guillemot et al., 2012).

### Clinical Application

Plerixafor (AMD3100) is a CXCR4 antagonist that was formally approved by the US FDA in 2008 to use in combination with autologous transplantation in patients with Non-Hodgkin’s Lymphoma or multiple myeloma (De Clercq, 2019). By blocking the interaction between CXCL12 and CXCR4, plerixafor triggers the mobilization of stem and progenitor cells (CD34^+^ cells) (DiPersio et al., 2009). These stem cells are then collected and used in autologous stem cell transplantation to rescue the hematopoietic toxicity and to reconstitute hematopoiesis following high-dose chemotherapy. Furthermore, plerixafor injection is used in combination with a granulocyte-colony stimulating factor (G-CSF) medication to prepare the blood for an autologous stem cell transplant. A clinical study involved patients with non-Hodgkin’s lymphoma or multiple myeloma showed the combination of plerixafor and G-CSF was superior to G-CSF alone in mobilizing hematopoietic progenitor cells (Flomenberg et al., 2005). Recent clinical trials indicate that plerixafor could be used in other strategies for treating cancer. A phase 1/2 study indicated that the addition of plerixafor to cytotoxic chemotherapy could increase the rates of remission in acute myeloid leukemia (Uy et al., 2012). Similarly, the combination of radiochemotherapy (RTCT) and plerixafor yielded a greater delay in tumor growth and lymph node metastasis in patients with cervical cancer than did RTCT alone (Chaudary et al., 2017). Moreover, the combination of plerixafor and bortezomib yielded a strong response rate in relapsed/refractory multiple myeloma (Ghobrial et al., 2019). According to the clinical studies, combination with other current cancer therapies would be the primary application of plerixafor or other CXCR4 antagonists. Therefore, pharmacokinetic studies and rational design of dosage are definitely required to avoid potential side effects.

### Cancer Immunotherapy

According to the major role of CXCL12 axis in TME, lots of antagonists targeting CXCL12axis were proposed as monotherapy to promote antitumor immunity or in combination with other immunotherapies for cancer treatment. In a mouse model of ovarian cancer, AMD3100 treatment showed significant increases in T-cell–mediated antitumor immune responses, resulting in CXCR4 positive tumor apoptosis and necrosis (Righi et al., 2011). Similar results were obtained in multiple cancer models, that AMD3100 administration leads to rapid T cell accumulation and acted synergistically with immunological checkpoint antagonists (anti-PD-L1) (Feig et al., 2013; Chen et al., 2015). AMD3100 also increases the efficiency of the mesothelin-targeted immune-activating fusion protein (VIC-008) in mesothelioma, which is regulated by PD-1suppression in CD8+T cells and conversion of regulatory T into helper-like cells (Li et al., 2018). Another study focused on chemoresistant ovarian cancer reported a novel oncolytic vaccinia virus expressing a CXCR4 antagonist that can reduce the immunosuppressive network and increase tumor apoptosis and phagocytosis alone or in combination with doxorubicin (Komorowski et al., 2016). Genget al. developed a new FAPα-targeted vaccine for the treatment of breast cancer (Geng et al., 2019). This DNA vaccine enhanced antigen secretion and effectively decreased the number of CAFs in the TME, leading to decreases in CCL2 and CXCL12 expression, thereby reducing the myeloid-derived suppressor cells in the TME. In glioblastoma, CXCR7-targeted antibody (X7Ab) enhanced tumor cell phagocytosis by increasing macrophages activity (Salazar et al., 2018). And combining X7Abwith Temozolomide (TMZ) significantly slowed mouse glioma progression with prolonged survival. Cancer immunotherapy targeting CXCL12 axis are giving encouraging results. Further clinical studies based on these findings should be performed to increase the effectiveness of cancer therapy.

## Conclusion

Compelling evidence has demonstrated that CXCL12/CXCR4/CXCR7 axis is implicated in tumor growth, survival, angiogenesis, metastasis, tumor microenvironment, and chemoresistance. Thus, the CXCL12 axis is a promising target for therapeutic intervention. However, only a few drugs that target the CXCL12 axis have been approved for clinical use. CXCL12 and its receptors play important roles in homeostasis and non-pathologic inflammation, which might predict that agents that target the CXCL12 axis would possess significant on-target toxicities. Furthermore, crosstalk between CXCR4 and CXCR7 makes specific CXCR4 targeting more challenging. Relatively little is known about the function of CXCR7 and its signal transduction in cancer genesis and/or progression. Elucidating these functions and their mechanisms will undoubtedly contribute to the development of better anticancer agents that target the CXCL12 axis. In clinical applications, the development of targeted drug delivery systems for CXCR4 antagonists should be considered to increase the efficacy of these therapies and to reduce their side effects.

## Author Contributions

YS collected the original materials and wrote the first draft. DJR and JS made significant changes on the scope and format of this review.

## Funding

This study was supported partially by NIH funding 1R01HL125279-01A1 (JS).

## Conflict of Interest

The authors declare that the research was conducted in the absence of any commercial or financial relationships that could be construed as a potential conflict of interest.
